# Neuromuscular Blockade for Intracranial Hypertension: Quantifying Real-World Effectiveness on Intracranial Pressure

**DOI:** 10.1177/08850666261432119

**Published:** 2026-03-16

**Authors:** Noam Goder, Gordon D. Rubenfeld, Andre C.K.B. Amaral

**Affiliations:** 1 Department of Critical Care Medicine, 71545 Sunnybrook Health Sciences Centre, Toronto, Ontario, Canada; 2 Interdepartmental Division of Critical Care Medicine, University of Toronto, Toronto, Ontario, Canada

**Keywords:** rocuronium, neuromuscular blockade, intracranial pressure, neurocritical care, traumatic brain injury

## Abstract

**Background:**

Contemporary intracranial hypertension guidelines, including SIBICC (2019) and the 2024 ACS Best Practices, list neuromuscular blockade as a Tier Two, short-duration option for refractory ICP elevation. Despite its physiologic rationale, the real-world effect of initiating continuous paralysis on ICP has not been quantified using large-scale intra-patient physiologic data.

**Methods:**

We performed a retrospective intra-patient before–after study at a Level 1 trauma and neurocritical care center (2016-2021). Among 9373 ICU admissions, 106 patients receiving a Rocuronium infusion with continuous invasive ICP monitoring met inclusion criteria. ICP values were compared using symmetric pre/post windows (−165-0 min; +15-180 min). A generalized additive model (GAM) evaluated time-dependent ICP changes adjusting for sedatives and hypertonic saline.

**Results:**

Median ICP decreased from 20.7 to 18.6 mm Hg (within-patient Δ −0.9 mm Hg; *P* = .017). Cerebral perfusion pressure increased from 71.1 ± 10.5 to 75.2 ± 12.5 mm Hg (*P* < .001). In patients with pre-treatment ICP > 20 mm Hg, median ΔICP was −2.0 mm Hg (*P* < .001). Adjusted GAM modeling demonstrated reductions of −1.66 mm Hg at 3 h and −2.23 mm Hg at 6 h.

**Conclusions:**

Continuous Rocuronium infusion was associated with modest (approximately 1-2 mm Hg) reductions in ICP and improved CPP. Although these physiologic changes are small and may have limited clinical significance, this study provides the largest real-world evaluation and quantification of the effect of continuous neuromuscular blockade on ICP control and directly informs a major evidence gap highlighted in contemporary guidelines.

## Background

Intracranial hypertension (ICH) is a significant contributor to morbidity and mortality among neurocritical care patients. The management of elevated intracranial pressure (ICP) is critical in mitigating secondary brain injury and improving patient outcomes.^1,2^ Neuromuscular blockade agents (NMBA), such as Rocuronium, are frequently employed to manage acute elevations in ICP during routine intensive care procedures—including tracheal suctioning, physiotherapy, and episodes of patient agitation—all of which can provoke transient spikes in ICP.^3–5^.

## Cohort Studies Evaluating NMBA and Intracranial Pressure

Several cohort studies have examined the effects of NMBA on ICP by comparing outcomes across patient groups with differing sedative and paralytic regimens. Kerr et al conducted a prospective cohort study in 71 adults with severe traumatic brain injury (TBI) undergoing standardized endotracheal suctioning. Patients were allocated to one of three treatment strategies: no medication, opioids alone (morphine sulfate or fentanyl citrate), or opioids combined with the non-depolarizing NMBA vecuronium. Those receiving both opioids and vecuronium exhibited significantly smaller increases in ICP during suctioning compared to the other groups, although no difference was observed in the rate of return to baseline ICP.^6^

A systematic review by Sanfilippo et al^7^ summarized 22 prospective studies and highlighted that NMBA boluses were consistently effective in blunting ICP surges during procedural stimulation—particularly endotracheal suctioning and physiotherapy. However, retrospective cohort studies have raised concerns about the broader application of continuous NMBA use. In a secondary analysis of a multicenter trial, Juul et al found that patients treated with NMBA infusions spent a significantly greater number of hours with ICP > 20 mm Hg compared to those not receiving NMBA (13.5 vs 6.5 h, *P* < .05).^8^ Similarly, Hsiang et al reported that early continuous paralysis (>12 h) was not associated with improved neurologic outcomes, but was linked to prolonged ICU stay and higher rates of pneumonia.^9^ These cohort findings suggest that while NMBAs may be beneficial for procedural control of ICP, their prolonged use may confer no lasting benefit and introduce additional risk.

## Intra-Patient Studies Evaluating NMBA and Intracranial Pressure

More definitive physiologic insight into the effect of NMBAs on ICP is derived from intra-patient studies, in which each subject serves as their own control. These designs are particularly valuable in minimizing inter-patient variability and confounding. In a prospective observational study, Gemma et al analyzed 131 episodes of endotracheal suctioning in 17 sedated, mechanically ventilated patients with severe head injury. They found that ICP increased modestly (+2 mm Hg) in patients who remained immobile during suctioning but surged significantly (+13 mm Hg) in those who coughed or moved.^10^ A similar intra-patient crossover design was employed by Werba et al in a randomized study of 18 neurosurgical ICU patients with GCS < 7. In all cases, tracheal suctioning without muscle relaxation caused moderate to severe coughing and led to significant ICP elevations (from 18-19 to 24-27 mm Hg) and CPP reductions. After bolus administration of vecuronium or atracurium, suctioning induced no significant change in ICP or CPP, confirming that deep neuromuscular blockade suppressed the diaphragmatic movement responsible for these hemodynamic spikes.^11^ Schramm et al conducted an intra-patient study in 20 sedated neurosurgical ICU patients, comparing ICP and CPP before and after administration of rocuronium or vecuronium. Neither agent caused significant changes in ICP or CPP, confirming that under stable conditions, non-depolarizing NMBAs do not adversely affect cerebral dynamics.^5^

## Current Recommendations

Despite physiologic evidence that neuromuscular blockade can blunt procedure-related ICP surges, major societies either make no specific recommendation for or against NMBAs or classify them as a second-tier option rather than a routine first-line therapy for ICP control. The 2016 SCCM guidelines state that, among adults with acute brain injury and elevated ICP, “we make no recommendations as to whether neuromuscular blockade is beneficial or harmful” because the available data are limited to two small observational studies.^12^ A review by Renew et al cautions that while short-term paralysis may reduce ICP by suppressing coughing or ventilator dyssynchrony, prolonged use carries risks such as ICU-acquired weakness and increased costs without proven outcome benefit.^13^ The 2019 Seattle International Brain Injury Consensus Conference (SIBICC) three-tier algorithm classifies neuromuscular blockade as a Tier Two intervention for refractory intracranial hypertension and recommends a short, efficacy-based trial in adequately sedated patients, with continuation only if ICP reduction is demonstrated and discontinuation as soon as feasible.^14^ The 2024 American College of Surgeons Best Practices Guidelines: The Management of Traumatic Brain Injury adopt this tiered framework, listing neuromuscular paralysis as a Tier Two option “in adequately sedated patients, if efficacious in lowering ICP,” and advising initiation with a trial dose and escalation to continuous infusion only when benefit is evident, within a structured reassessment of overall ICP management.^15^

Together, these recommendations support selective, time-limited NMBA use in patients with uncontrolled ICP despite optimal sedation, while discouraging indiscriminate or prolonged paralysis.

## Rationale and Study Aim

To overcome the limitations of previous studies, we took advantage of our electronic patient record which records ICP in a real-world clinical environment. The study was designed to understand the immediate effects of starting a neuromuscular blockade infusion on ICP trajectories, rather than to compare outcomes with other treatment strategies or no intervention.

## Methods

### Study Design and Setting

This was a retrospective intra-patient time-series study conducted at Sunnybrook Health Sciences Centre, a Level 1 Trauma and Neurocritical Care Center in Toronto. The study spanned from 2016 to 2021 and evaluated high-resolution physiologic changes surrounding Rocuronium administration.

ICP monitors were placed according to Brain Trauma Foundation guidelines and were almost exclusively intraparenchymal. A formal escalation ladder for managing elevated intracranial pressure (ICP), loosely based on Brain Trauma Foundation (BTF) guidelines, is followed in clinical practice. This typically includes head-of-bed elevation, collar adjustment, osmotherapy (usually hypertonic saline), and deep sedation with agents such as propofol or midazolam. Rocuronium is the only neuromuscular blocker used. Physiologic and clinical data were extracted from the electronic medical record, which integrates continuous vital-signs monitoring with nurse-verified or manually entered documentation.

### Patient Population

Eligible patients were those admitted to the Trauma and Neurotrauma Intensive Care Unit, which manages both general trauma and neurocritical care patients, who received any infusion of Rocuronium and underwent continuous invasive ICP monitoring. Single boluses of rocuronium were not captured as they did not address our research question. To be included, patients were required to have at least one valid ICP measurement between 165 and 0 min prior to Rocuronium administration, and at least one between 15 and +180 min following the dose. Implausible ICP values (≤0 or >50 mm Hg) were removed, but patients were retained in the analysis if valid ICP measurements remained both before and after Rocuronium administration.

### Exposure and Outcomes

The exposure of interest was the start time of the first infusion of rocuronium. The primary outcome was the change in average intracranial pressure (ICP), calculated from values recorded before and after Rocuronium administration. Secondary analyses were conducted in two predefined subgroups: (1) patients with a maximum ICP > 20 mm Hg in the 165 min preceding Rocuronium, and (2) patients who did not receive hypertonic saline within ±60 min of Rocuronium administration.

### Data Sources and Processing

Data were extracted from the electronic medical record using the MetaVision system (iMDsoft, Tel Aviv, Israel). Intracranial pressure (ICP) values are transmitted directly from the bedside monitor to MetaVision. In contrast, the timing of intravenous infusions—including Rocuronium—is entered manually by the nurse at the bedside.

During the study period, intracranial pressure (ICP) was validated and stored as hourly measurements for all patients. In addition, a subset of patients had high-frequency, minute-by-minute data available, representing automatically captured values that were not manually validated by nurses.

The time of the first Rocuronium infusion was used as the reference point. For the primary analysis, intracranial pressure (ICP) values were standardized to hourly resolution to ensure uniform sampling across patients. When minute-by-minute data were available, values were averaged within each hour. ICP was analyzed within symmetric windows extending from 165 min before to 180 min after Rocuronium administration. Pre-treatment ICP was defined as the mean of hourly values between −165 and 0 min, and post-treatment ICP as the mean between +15 and +180 min. The 15-min offset allowed sufficient time for the drug to take effect. In a sensitivity analysis, all calculations were repeated using the original minute-by-minute data to confirm consistency of results.

Variables collected included intracranial pressure (ICP), cerebral perfusion pressure (CPP), mean arterial pressure (MAP), end-tidal CO_2_ (ETCO_2_), peak inspiratory pressure (PIP), and arterial blood gas values including pCO_2_. Additional data included norepinephrine dose, use of sedative and analgesic medications (propofol, midazolam, fentanyl, ketamine, hydromorphone), timing of maximum ICP prior to Rocuronium, and exposure to hypertonic saline within ±60 min. Demographic and clinical characteristics such as age, sex, admission diagnosis, initial GCS, hospital length of stay, and in-hospital mortality were also recorded.

The study was approved by the institutional review board (number 3690).

### Statistical Analysis

#### Descriptive and Comparative Analyses

Continuous variables were summarized using mean ± standard deviation (SD) for normally distributed data and median with interquartile range (IQR) for non-normally distributed data. Categorical variables were reported as counts and percentages. Paired t-tests were used for comparisons involving normally distributed paired data, while non-parametric alternatives were applied for skewed distributions. The Wilcoxon signed-rank test was used to compare paired pre- and post-treatment values when distributions were non-normal. Spearman's correlation coefficient was used to evaluate monotonic associations between changes in continuous variables. All statistical tests were two-tailed, and a *P*-value < .05 was considered statistically significant.

#### GAM Modeling

We evaluated the effect of Rocuronium on intracranial pressure (ICP) using a generalized additive model (GAM) with patient-level random effects. ICP measurements were analyzed within a ± 24-h window centered on Rocuronium initiation. The model included a smooth function of time to capture nonlinear changes in ICP and a random effect for each patient to account for repeated measurements. The analysis was adjusted for concurrent sedative and analgesic infusions (propofol, midazolam, fentanyl, ketamine, hydromorphone) and for hypertonic saline administered within ±60 min. Model predictions were centered on the time of Rocuronium initiation so that changes in ICP represented differences relative to that point. Estimated effects with 95% confidence intervals were reported at 1, 3, and 6 h after Rocuronium administration.

#### Software

All analyses were performed using R version 4.5.0 (R Foundation for Statistical Computing, Vienna, Austria).

## Results

### Cohort Characteristics

From 2016 to 2021, 9373 patients were admitted to the Trauma/Neurotrauma ICU. A total of 632 patients had ICP data available in the assessment records. From this broader cohort, 106 (16.8%) cases were treated with a rocuronium infusion (Table 1). The median time from ICU admission to Rocuronium administration was 41.1 h (interquartile range [IQR] 20.6-78.0). The median duration of rocuronium infusion was 35.9 h (IQR 15.5-65.5). Patient age ranged from 19 to 80 years, with a median of 39 years. Males comprised 71.7% of the study population.

**Table 1.** Cohort Characteristics of Rocuronium-Treated Patients.

Characteristic	N = 106^a^
Age	39 [28, 53]
Sex	
F	30 / 106 (28%)
M	76 / 106 (72%)
Diagnosis	
Traumatic brain injury	80 / 106 (75%)
Subarachnoid hemorrhage	16 / 106 (15%)
Spontaneous intracranial hemorrhage	6 / 106 (5.7%)
Intracranial mass/tumor	1 / 106 (0.9%)
Hemorrhagic stroke	1 / 106 (0.9%)
Non-hemorrhagic stroke	1 / 106 (0.9%)
Other	1 / 106 (0.9%)
Neurosurgical surgery	48 / 106 (45.3%)
First GCS Total	3.00 [3.00, 8.00]
Time from Admission to Rocuronium (hr)	41 [21, 78]
Death	30 / 106 (28%)
Length of Stay (days)	26 [14, 40]
Weight (kg)	93 ± 36

^a^ Median [Q1, Q3]; n / N (%); Mean ± SD

The most common admitting diagnosis was traumatic brain injury (80 patients, 75.5%), followed by subarachnoid hemorrhage (16 patients, 15.1%) and spontaneous intracranial hemorrhage (6 patients, 5.7%). Less frequent diagnoses included intracranial mass or tumor (1 patient, 0.9%), hemorrhagic stroke (1 patient, 0.9%), non-hemorrhagic stroke (1 patient, 0.9%), and other (1 patient, 0.9%), as summarized in Table 1.

Overall, 48 patients (45.3%) underwent a neurosurgical cranial surgery (craniotomy or craniectomy). Of these, 36 (34.0%) had surgery prior to rocuronium initiation, while 12 (11.3%) underwent surgery after rocuronium administration.

### Pre/Post Rocuronium Analysis

In a descriptive analysis using symmetric time windows, median ICP prior to Rocuronium administration (−165 to 0 min) was 20.7 mm Hg (IQR 16.8-24.5), compared with 18.6 mm Hg (IQR 13.2-22.4) in the post-treatment period (+15 to +180 min), corresponding to a within-patient median reduction of −0.9 mm Hg (IQR −5.3 to 1.6; *P* = .017). The absolute peak intracranial pressure in the pre-treatment window was 24.0 mm Hg (IQR 19.0-29.1; range 3-49 mm Hg), typically occurring approximately −105 min before Rocuronium administration (IQR −165 to −45, Figure 1).

**Figure 1.**
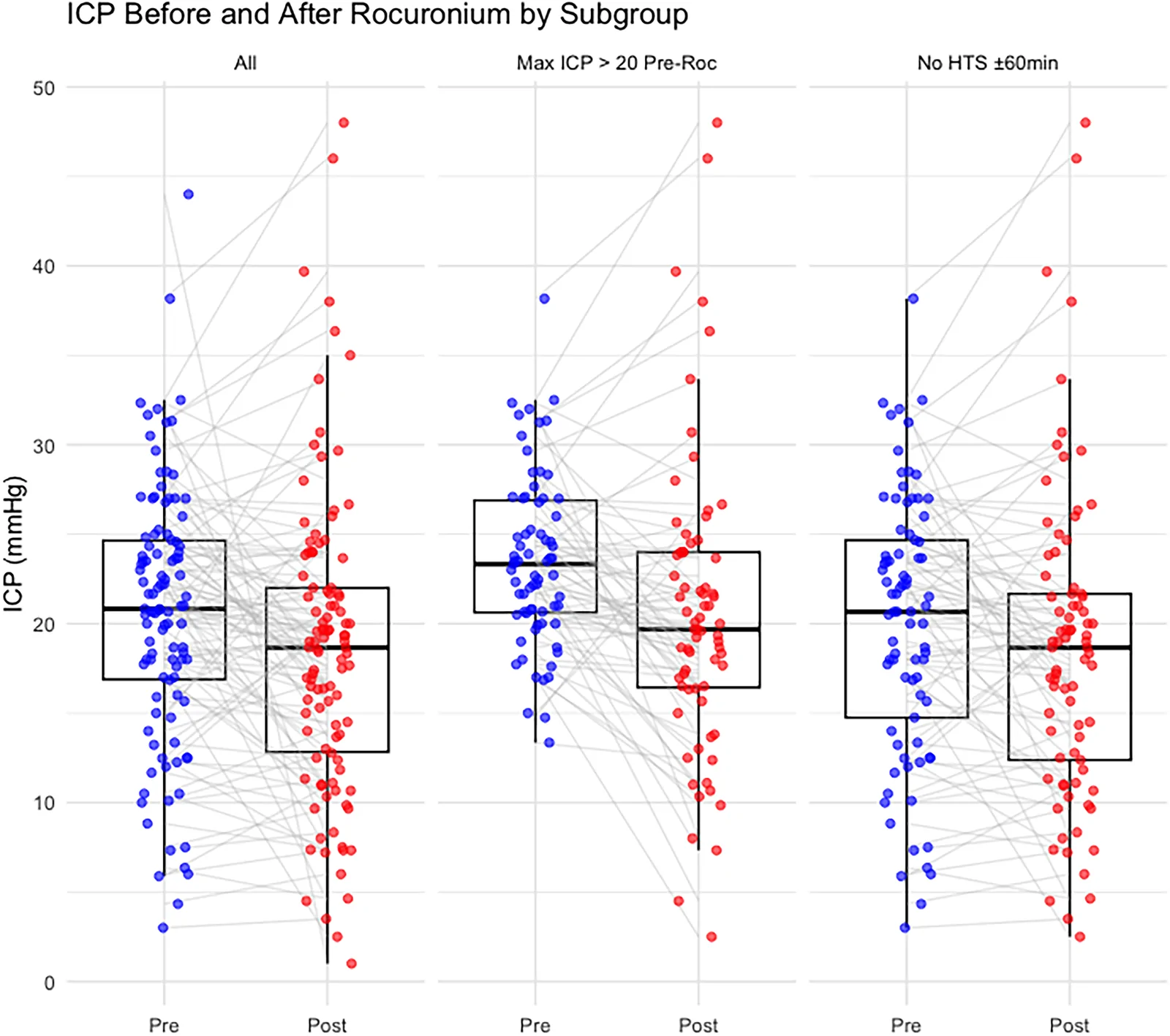
Intracranial pressure trajectories before and after initiation of continuous Rocuronium infusion.

When stratified by neurosurgical surgery timing, 36 patients underwent cranial surgery prior to rocuronium administration. In this group, the median within-patient ICP change was −0.37 mm Hg (IQR −3.25 to 1.33), which was not statistically significant (*P* = .22). In contrast, 70 patients did not undergo cranial surgery prior to rocuronium (including those without surgery or with surgery after starting rocuronium infusion). In this group, the median ICP change was −1.31 mm Hg (IQR −5.76 to 2.33), which reached statistical significance (*P* = .043).

**Sensitivity analysis:** When restricted to patients with minute-by-minute intracranial pressure measurements (n = 27), the median ICP decreased from 19.9 mm Hg (IQR 13.2-23.9) before treatment to 17.4 mm Hg (IQR 11.1-21.5) after treatment (*P* = .185). The corresponding within-patient median change was −0.97 mm Hg (IQR −3.03 to 0.98; *P* = .185).

### Hemodynamic and Ventilatory Effects of Rocuronium

Following Rocuronium administration, cerebral perfusion pressure (CPP) increased from 71.1 ± 10.5 to 75.2 ± 12.5 mm Hg (*P* < .001), while mean arterial pressure (MAP) increased from 91.8 ± 9.9 to 93.1 ± 10.6 mm Hg, a non-significant change (*P* = 0.15; Figure 2, Table 2).

**Figure 2.**
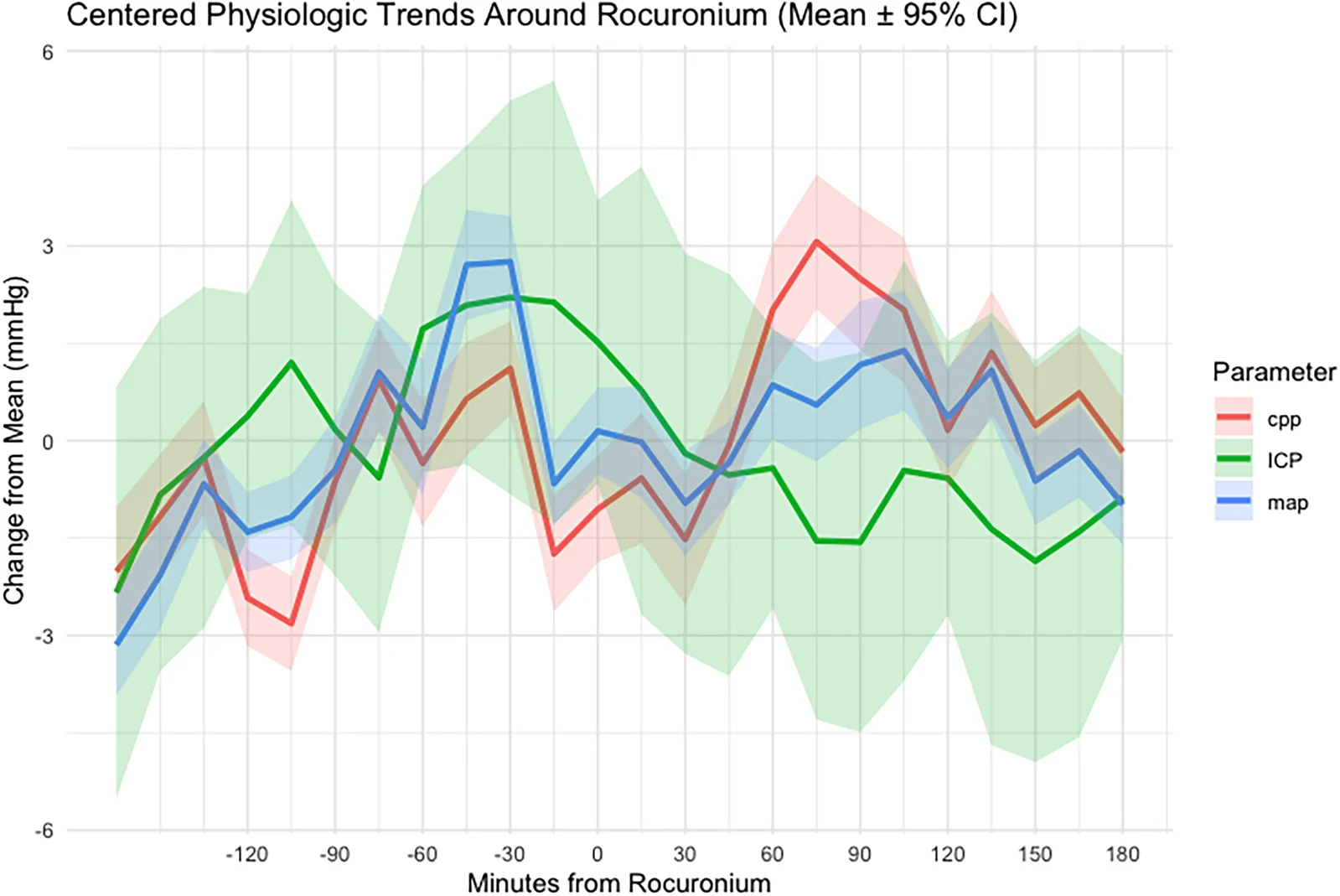
Time-aligned trajectories of intracranial pressure (ICP), mean arterial pressure (MAP), and cerebral perfusion pressure (CPP) following Rocuronium administration.

**Table 2.** Physiologic and Sedative Data Before and After Rocuronium.

Variable	Pre N = 106^a^	Post N = 106^a^	*P*-Value^b^
ICP	20.7 [16.8, 24.5]	18.6 [13.2, 22.4]	.017
CPP	71.1 ± 10.5	75.2 ± 12.5	<.001
MAP	91.8 ± 9.9	93.1 ± 10.6	.151
ETCO_2_	31.7 ± 6.3	30.1 ± 6.3	<.001
Peak inspiratory pressure	22.5 [19.0, 29.0]	22.0 [19.0, 28.0]	.738
pCO_2_	39.0 ± 8.3	38.5 ± 6.3	.549
Norepinephrine dose	10.0 [6.0, 20.0]	10.0 [6.0, 18.0]	.6

^a^ Median [Q1, Q3]; Mean ± SD

^b^ Paired t-test for means; Wilcoxon signed-rank for medians.

Units: ICP/CPP/MAP/ETCO_2_/pCO_2_ in mm Hg; Peak pressure in cm H_2_O. Norepinephrine µg/min. ETCO_2_ available in 85 patients; arterial pCO_2_ in 105 patients.

In 85 patients with complete ETCO_2_ and ICP data, mean ETCO_2_ decreased from 31.7 ± 6.3 to 30.1 ± 6.3 mm Hg (*P* < .001). Despite this reduction, there was no significant correlation between ETCO_2_ change and ICP change (Spearman's ρ = 0.12, *P* = .26).

Among 105 patients with arterial blood gas data, mean pCO_2_ decreased from 39.0 ± 8.3 to 38.5 ± 6.3 mm Hg (*P* = .549). Similarly, changes in pCO_2_ were not associated with changes in ICP (Spearman's ρ = −0.01, *P* = .92).

Sedative and analgesic exposure before and after Rocuronium administration is summarized in Table 3.

**Table 3.** Sedative Exposure and Dose Summary.

Sedative	Pre N (%)	Post N (%)	*P* (use)	Dose Pre [IQR]	Dose Post [IQR]	*P* (dose)
Propofol (mcg/kg/min)	87 (82.1%)	89 (84.0%)	.683	100 [75, 100]	100 [75, 100]	.709
Midazolam (mg/hr)	72 (67.9%)	87 (82.1%)	<.001	10.0 [10.0, 16.2]	10.0 [6.5, 15.0]	<.001
Fentanyl (mcg/hr)	87 (82.1%)	89 (84.0%)	.617	200 [150, 200]	200 [150, 200]	.117
Ketamine (mg/kg/hr)	14 (13.2%)	20 (18.9%)	.041	1.2 [1.0, 2.0]	1.0 [1.0, 2.0]	.022
Hydromorphone (mg/hr)	15 (14.2%)	16 (15.1%)	1.000	2.0 [1.0, 2.0]	2.0 [1.0, 2.0]	.371

### Subgroup Analyses

The effect of Rocuronium on ICP was further examined in two clinically relevant subgroups. In patients with elevated ICP prior to treatment, defined as a peak ICP greater than 20 mm Hg in the 165 min before Rocuronium, the median reduction was −2.0 mm Hg (IQR −7.7 to 0.5; *P* < .001). In contrast, among patients who did not receive hypertonic saline within ±60 min of Rocuronium administration, the median change was smaller and not statistically significant at −1.0 mm Hg (IQR −4.3 to 2.2; *P* = .13). For comparison, the response to hypertonic saline was analyzed separately across 466 episodes in this cohort and is presented in the Supplement.

### Generalized Additive Model of Intracranial Pressure

A generalized additive model (GAM) adjusting for concurrent administration of fentanyl, midazolam, propofol, ketamine, hydromorphone, and hypertonic saline within the preceding 60 min was applied to 7251 intracranial pressure (ICP) values within a ± 24-h window around rocuronium administration. The model estimated mean changes in ICP relative to time 0 of −0.55 mm Hg at 1 h (95% CI −1.76 to 0.65; *P* = .37), −1.66 mm Hg at 3 h (95% CI −2.88 to −0.44; *P* < .01), and −2.23 mm Hg at 6 h (95% CI −3.43 to −1.02; *P* < .01). The nadir of the predicted ICP curve occurred approximately 21 h after rocuronium administration, corresponding to a maximal reduction of −2.96 mm Hg (95% CI −4.18 to −1.75; *P* < .01).

Within the 0-6-h window, ICP showed a progressive decline, becoming statistically significant after 3 h (*P* < .01) and reaching its greatest decrease by 6 h (Figure 3).

**Figure 3.**
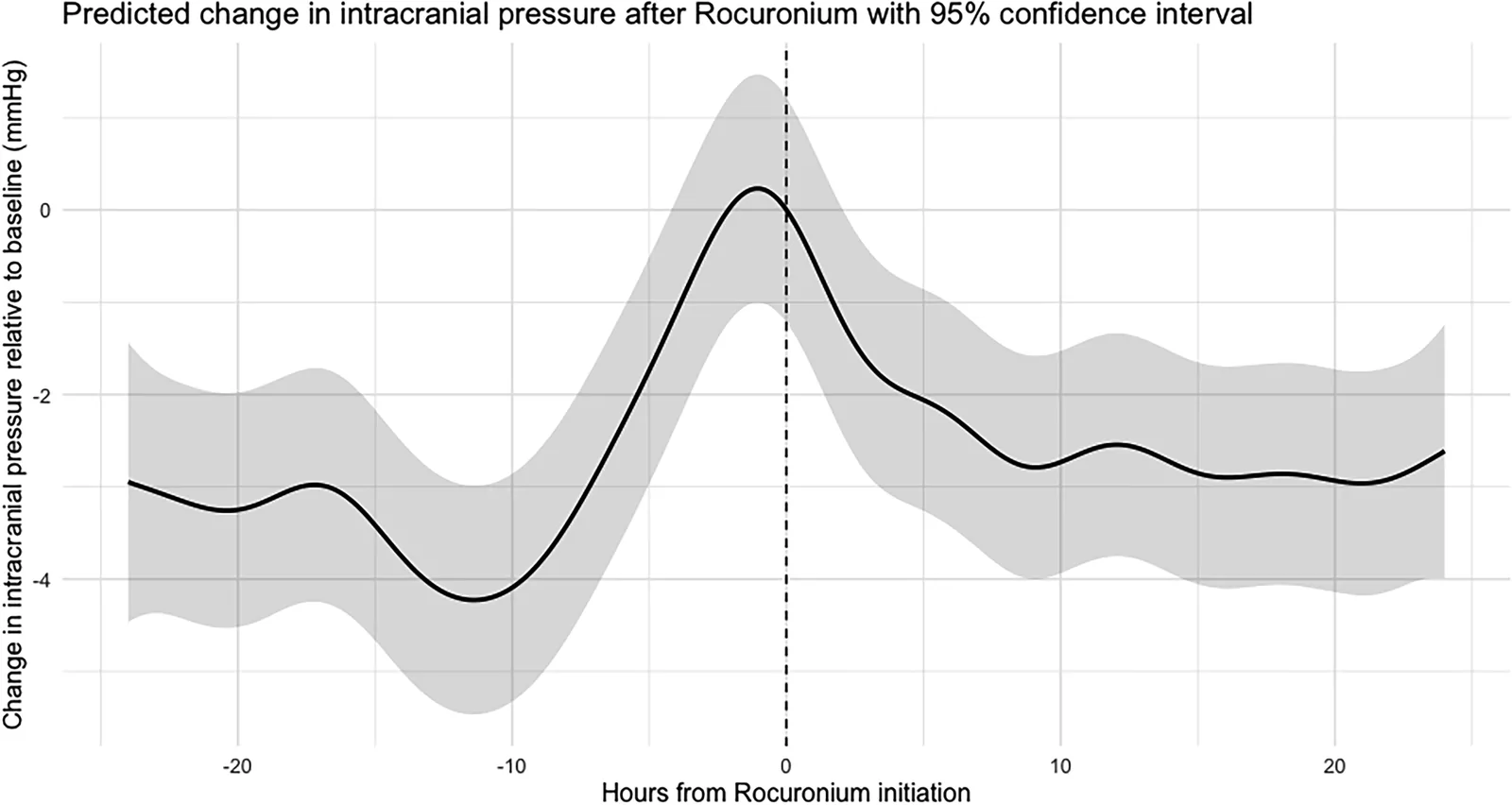
Generalized additive model (GAM) predicted change in intracranial pressure after Rocuronium, with 95% confidence intervals.

**Sensitivity analysis:** When restricted to patients with minute-by-minute measurements (n = 27), the model included 4149 measurements. Estimated changes in ICP relative to time 0 were −0.24 mm Hg at 1 h (95% CI −3.21 to 2.73; *P* = 0.88), −0.99 mm Hg at 3 h (95% CI −3.96 to 1.98; *P* = .51), and −1.82 mm Hg at 6 h (95% CI −4.79 to 1.16; *P* = .23). The predicted nadir occurred approximately 8 h after Rocuronium, corresponding to a maximal reduction of −2.01 mm Hg (95% CI −4.98 to 0.96; *P* = .19).

## Discussion

The main findings of our study are:

1. Rocuronium administration was associated with a modest but statistically significant reduction in intracranial pressure (ICP) among critically ill patients, with a median decrease of 0.9 mm Hg (IQR −5.3 to 1.6; *P* = .017). This signal was primarily observed in patients without prior cranial surgery, while those who underwent surgery before rocuronium did not demonstrate a significant ICP reduction.
2. The ICP-lowering effect was more pronounced in patients with elevated ICP prior to treatment; specifically, those experiencing an ICP peak greater than 20 mm Hg in the 165 min preceding Rocuronium showed a median ICP decrease of 2.0 mm Hg (IQR −7.7 to 0.5; *P* < .001).
3. Patients who did not receive hypertonic saline (HTS) within ±60 min of Rocuronium exhibited a smaller, non-significant median change of −1.0 mm Hg (IQR −4.3 to 2.2; *P* = .13), suggesting attenuation in lower-risk settings or a partial contribution of HTS to the full cohort effect.
4. Cerebral perfusion pressure (CPP) increased following Rocuronium administration, from 71.1 ± 10.5 to 75.2 ± 12.5 mm Hg; *P* < .001.
5. Generalized additive model (GAM) analysis demonstrated a modest time-dependent reduction in ICP, becoming statistically significant several hours after Rocuronium administration and persisting thereafter. The average reduction during this period was approximately 1.5-2 mm Hg.

Several potential confounding factors were examined to clarify the observed ICP changes. Notably, a significant reduction in end-tidal CO_2_ (ETCO_2_) occurred following Rocuronium administration; however, the absence of a significant correlation between ETCO_2_ changes and ICP reduction suggests that the observed ICP effects were not primarily mediated through ventilatory adjustments. Further, arterial carbon dioxide (pCO_2_) and peak inspiratory pressures remained stable, indicating Rocuronium's ICP-lowering effect is likely independent of alterations in ventilation or sedation depth.

While the observed ICP reductions were modest, they may carry clinical relevance as part of comprehensive, multimodal strategies for managing intracranial hypertension. These results align with prior cohort studies that reported limited long-term benefits from continuous neuromuscular blockade but recognized potential advantages in short-term ICP control during acute episodes. Current guidelines emphasize cautious, individualized use of neuromuscular blockers for ICP management, specifically advocating targeted, brief interventions rather than prolonged administration.

In our cohort, the magnitude of ICP reduction appeared greater among patients with elevated pre-treatment ICP and among those who had not undergone prior cranial decompression. Although exploratory, this observation raises the possibility that neuromuscular blockade may be most physiologically relevant in patients with intact cranial vaults in whom coughing, ventilator dyssynchrony, or episodic ICP variability contribute to intracranial hypertension. Conversely, in patients who have undergone decompressive craniectomy or who exhibit low baseline ICP, the measurable impact of paralysis may be limited. These subgroup findings should be interpreted cautiously and are hypothesis-generating, but they may help inform individualized clinical decision-making.

The novelty of our study lies in its robust, real-time, partially high-resolution data acquisition, capturing continuous physiological data surrounding Rocuronium administration, and the relatively larger sample size compared to previous intra-patient studies. Unlike earlier research limited to smaller cohorts or procedural contexts, our methodology enabled a more detailed assessment of immediate, physiologic responses to Rocuronium in typical ICU conditions. Importantly, the use of generalized additive models (GAM) provided a novel analytic approach, allowing us to capture the dynamic, nonlinear evolution of ICP over time while accounting for concurrent therapies. Together, these features enhance both the internal validity and clinical applicability of our findings.

Hypertonic saline (HTS) was an important comparator in our analysis. It provided a useful reference for contextualizing Rocuronium's magnitude and timing of effect, while also serving as a key confounder. Given that HTS is often co-administered in patients with rising ICP, subgroup analyses excluding patients who received HTS within ±60 min of Rocuronium were essential to isolate the drug's direct contribution. This approach strengthened the internal validity of our findings while acknowledging the complex interplay of concurrent ICP-directed therapies.

This study has several limitations. As a retrospective single-center investigation, it inherently limits causal inference and generalizability. Additionally, approximately 20% of patients exhibited baseline ICP levels below 15 mm Hg, suggesting alternative indications for Rocuronium administration that could have diluted the observed effects. We addressed this limitation through focused subgroup analyses; however, residual confounding may persist. Another limitation is that ICP and medication timing data relied on manual nurse entry, potentially introducing inaccuracies or documentation delays. Importantly, many patients are likely to have received Rocuronium boluses immediately prior to infusion, but this information is not documented in the MetaVision dataset, limiting precision in exposure definition. Only 27 patients had minute-by-minute ICP data, which demonstrated a similar magnitude of ICP reduction but did not reach statistical significance, likely reflecting the smaller sample size. Finally, the absence of a control group further limits causal inference, and definitive attribution of causality will require prospective or comparative studies.

In conclusion, Rocuronium administration was associated with modest reductions in ICP, typically in the range of 1-2 mm Hg, with corresponding improvements in CPP. Given the small magnitude of these changes, the clinical significance is likely limited. However, this study provides valuable real-world, intra-patient quantification of the physiologic response to continuous neuromuscular blockade. These findings support its selective, short-duration use for refractory ICP elevation and underscore the need for prospective, comparative studies to clarify when, and in whom, paralysis offers meaningful benefit.

## Supplemental Material

sj-docx-1-jic-10.1177_08850666261432119 - Supplemental material for Neuromuscular Blockade for Intracranial Hypertension: Quantifying Real-World Effectiveness on Intracranial Pressure Supplemental material, sj-docx-1-jic-10.1177_08850666261432119 for Neuromuscular Blockade for Intracranial Hypertension: Quantifying Real-World Effectiveness on Intracranial Pressure by Noam Goder, Gordon D. Rubenfeld and Andre C.K.B. Amaral in Journal of Intensive Care Medicine

## Data Availability

The study was approved by the institutional review board (number 3690).
