# Supplementary material for: Neuromuscular Blockade for Intracranial Hypertension: Quantifying Real-World Effectiveness on Intracranial Pressure
Source: J Intensive Care Med. 2026 Mar 16;41(11):1105–12. doi: 10.1177/08850666261432119 (PMC13672926; doi:10.1177/08850666261432119)
Supplement: Supplementary file 1 [file sj-docx-1-jic-10.1177_08850666261432119.docx]

**Supplementary Results**

### Tracheostomy and PEG Placement

Tracheostomy was identified in 15 patients (14.2%), PEG placement in 10 patients (9.4%), and either procedure in 20 patients (18.9%) of the cohort based on free-text review. Rocuronium infusion duration did not significantly differ according to these procedures. Median infusion duration was 36.0 hours (IQR 12.0–64.2) among patients with tracheostomy compared with 35.9 hours (IQR 17.1–65.3) in those without (p = 0.856). Similarly, infusion duration did not differ in patients with versus without PEG placement (37.8 vs. 35.4 hours; p = 0.871), nor when considering the composite outcome of tracheostomy or PEG (36.8 vs. 35.4 hours; p = 0.888). These findings suggest that Rocuronium infusion duration was not associated with subsequent need for airway or enteral access procedures in this cohort.

### late 6-hour window

Among 104 patients with sufficient ICP data extending to 6 hours post-Rocuronium, median ICP during the late period (+195 to +360 minutes) remained reduced at 17.9 mmHg (IQR 12.2–21.1), corresponding to a decline of −2.0 mmHg from baseline (IQR −5.7 to 1.7; p = 0.0007). The difference between the early (+15 to +180 minutes) and late (+195 to +360 minutes) post-treatment windows was not statistically significant (−0.1 mmHg, IQR −2.5 to 1.7; p = 0.443).

### Intracranial Pressure Response to Hypertonic Saline

We examined the effect of hypertonic saline (HTS) on intracranial pressure (ICP) in patients who had received Rocuronium during their ICU stay. ICP trajectories were aligned to the time of HTS administration using a fixed window from 60 minutes before to 75 minutes after dosing. A total of 466 HTS episodes across 67 patients were included.

Median ICP declined from 20.0 mmHg (IQR 15.3–23.4) before treatment to 16.1 mmHg (IQR 12.0–20.4) after HTS. The peak ICP in the 60 minutes prior to administration was 21.0 mmHg (IQR 17.0–25.0). A linear mixed-effects model, accounting for repeated measures within patients, estimated a mean ICP reduction of −3.10 mmHg (95% CI −3.95 to −2.25; p < 0.001).

**Supplementary Figure S1-**Time-aligned ICP trajectories around hypertonic saline administration in Rocuronium-treated patients.

**Multivariable Analysis of Post-Rocuronium ICP**
 We performed a multivariable linear regression (ANCOVA) to evaluate predictors of ICP after Rocuronium administration. The dependent variable was the first post-treatment ICP, with baseline ICP and other pre-treatment variables included as covariates (baseline ICP, mean ETCO₂, propofol use, peak inspiratory pressure, norepinephrine dose, and MAP).

Baseline ICP was strongly associated with post-treatment ICP (β = 0.58, p < 0.001). Norepinephrine dose showed a non-significant trend toward higher ICP (β = 0.16, p = 0.11). Other covariates, including ETCO₂, propofol exposure, peak inspiratory pressure, and MAP, were not significantly associated with post-treatment ICP.

The model explained a modest proportion of variance (adjusted R² = 0.24) but was statistically significant overall (F(6,62) = 4.55, p < 0.001). Full regression coefficients are provided in Supplementary Table S1.

**Supplementary Figure S1-**Time-aligned ICP trajectories around hypertonic saline administration in Rocuronium-treated patients

### **Supplementary Table S1**

**Multivariable linear regression (ANCOVA) of predictors of post-Rocuronium ICP**

| **Predictor** | **Estimate (β)** | **Std. Error** | **t value** | **p value** |
| --- | --- | --- | --- | --- |
| Baseline ICP | 0.58 | 0.14 | 4.21 | <0.001 |
| Baseline ETCO₂ | 0.01 | 0.14 | 0.06 | 0.95 |
| Propofol at Rocuronium | −2.55 | 2.79 | −0.91 | 0.36 |
| Peak inspiratory pressure | −0.10 | 0.13 | −0.79 | 0.43 |
| Norepinephrine dose | 0.16 | 0.10 | 1.64 | 0.11 |
| Baseline MAP | −0.09 | 0.11 | −0.77 | 0.45 |
